# Risk factors and predictive model for pulmonary complications in patients transferred to ICU after hepatectomy

**DOI:** 10.1186/s12893-023-02019-1

**Published:** 2023-06-03

**Authors:** Bin Wang, HanSheng Liang, HuiYing Zhao, JiaWei Shen, YouZhong An, Yi Feng

**Affiliations:** 1 Department of Critical Care Medicine, Peking University People’s Hospital, No.11 Xizhimen South Street, Beijing, 100044 China; 2 Department of Anaesthesiology and Pain Medicine, Peking University People’s Hospital, No.11 Xizhimen South Street, Beijing, 100044 China

**Keywords:** Risk factor, Postoperative pulmonary complication, Nomogram, Hepatectomy

## Abstract

**Objective:**

Postoperative pulmonary complications (PPCs) seriously harm the recovery and prognosis of patients undergoing surgery. However, its related risk factors in critical patients after hepatectomy have been rarely reported. This study aimed at analyzing the factors related to PPCs in critical adult patients after hepatectomy and create a nomogram for prediction of the PPCs.

**Methods:**

503 patients’ data were collected form the Peking University People’s Hospital. Multivariate logistic regression analysis was used to identify independent risk factors to derive the nomogram. Nomogram’s discriminatory ability was assessed using the area under the receiver operating characteristic curve (AUC), and calibration was assessed using the Hosmer–Lemeshow goodness-of-fit test and calibration curve.

**Results:**

The independent risk factor for PPCs are advanced age (odds ratio [OR] = 1.026; P = 0.008), higher body mass index (OR = 1.139; P < 0.001), lower preoperative serum albumin level (OR = 0.961; P = 0.037), and intensive care unit first day infusion volume (OR = 1.152; P = 0.040). And based on this, we created a nomogram to predict the occurrence of PPCs. Upon assessing the nomogram’s predictive ability, the AUC for the model was 0.713( 95% CI: 0.668–0.758, P<0.001). The Hosmer–Lemeshow test (P = 0.590) and calibration curve showed good calibration for the prediction of PPCs.

**Conclusions:**

The prevalence and mortality of postoperative pulmonary complications in critical adult patients after hepatectomy are high. Advanced age, higher body mass index, lower preoperative serum albumin and intensive care unit first day infusion volume were found to be significantly associated with PPCs. And we created a nomogram model which can be used to predict the occurrence of PPCs.

## Introduction

Postoperative pulmonary complications (PPCs) adversely influence the mortality and hospital stays of patients after upper abdominal surgery [1, 2]. Their incidence after abdominal surgery ranges from 10 to 80% [3, 4]. Hepatectomy is one kind of common upper abdominal surgery, performed close to the diaphragm, and patients undergo this surgery often complicated with liver insufficiency and hypoproteinemia [5], these lead to higher incidence of PPCs in this kind of patients. Meanwhile, with the development of critical care medicine and the increase quantity of surgeries, more and more critical patients are transferred to the ICU after hepatectomy. These patients have more complex complications and more likely to develop the PPCs [6, 7].

Several studies reporting the risk factors of pulmonary complications after hepatectomy, including a few multivariate analyses, are usually limited in those non-critical patients [4, 8, 9]. A small number of studies have looked at critically ill populations, but these studies have small sample sizes and just included few variables, and most do not contain detailed information about intraoperative anesthesia or surgery [5, 10].

To the best of our knowledge, there have been no high-quality studies on the risk of pulmonary complications after hepatectomy in the critically ill population and no study have created a nomogram for prediction of the PPCs in these people. And the analysis of the factors related to the occurrence of PPCs in critical patients and build a prediction model will have great clinical value in establishing a feasible path for clinical intervention to reduce the occurrence of PPCs in this kind of patients. In this study, data from a 7-year clinical database were retrospectively analyzed, aimed to determine the risk factors for PPCs of critical patients after hepatectomy and build a nomogram model for the prediction of PPCs.

## Methods

### Study design and objectives

In this retrospective study, we sought to analyze the risk factors for PPCs and build a nomogram model for the prediction of PPCs. The study was approved by the local Ethics Committee, Peking University People’s Hospital, China (No. 2021PHB024-001). The study was conducted in the Peking University People’s Hospital, Beijing, China.

**Fig. 1:**
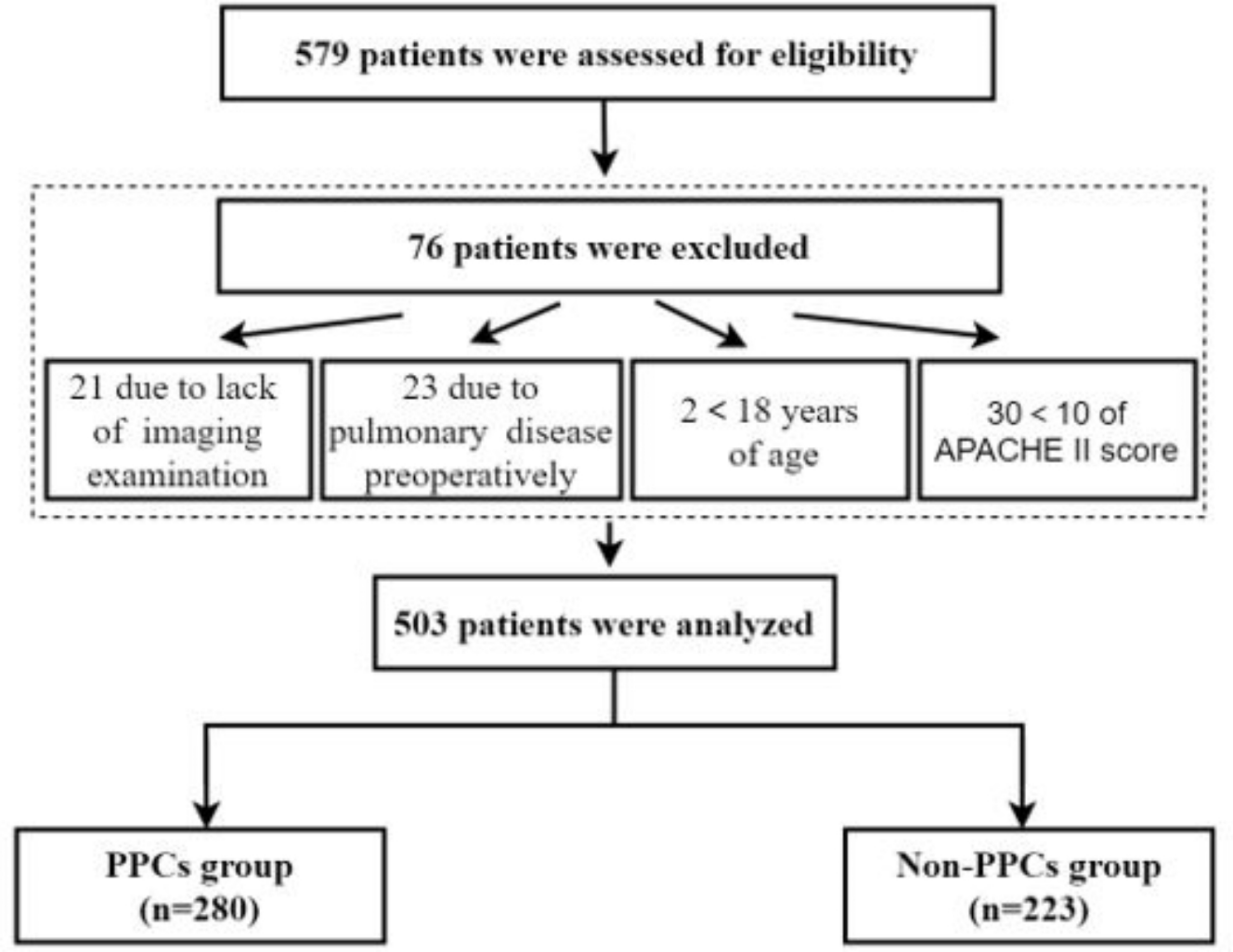
Study Flow

Patients admitted to the ICU after hepatectomy at Peking University People’s Hospital between January 2014 and December 2021 were considered eligible for this study. And the ICU admission standard for patients in our hospital are: 1.The patient was older than 75 years; 2. Patients with unstable control of complications or symptoms; 3. Intraoperative bleeding greater than 1000ml; 4. Patients need large dose of vasoactive drugs during surgery; 5. Patients with decreased oxygenation or difficulty in removing tracheal intubation during or after the operation; 6. Emergency events occur during the perioperative period; 7. Patients with high risk of postoperative bleeding or complications considered by surgeon(Those who meet one or more of the above criteria can be considered to transfer to ICU). Patients had an Acute Physiology and Chronic Health Evaluation II (APACHE II) score ≥ 10 were defined as critical patients [11, 12]. The patients’ data were retrospectively analyzed. The exclusion criteria were patients ≤ 18 years, those with preoperative pulmonary infection or other pulmonary diseases or the APACHEII score<10 (Fig. 1). Pulmonary diseases include history of chronic obstructive pulmonary disease, asthma, chronic bronchitis, lung surgery, chest trauma, etc. And, patients with pulmonary auscultation murmur, cough, phlegm and chest tightness complaints during preoperative routine examination or patients with preoperative oxygen saturation monitoring < 93% will be excluded from the study. We recorded information in an electronic anesthetic documentation system certified by the Healthcare Information and Management Systems Society, which was rated stage 7 by Electronic Medical Records Adoption Model (HIMSS EMRAM7). All data were obtained from the electronic database on the server. The objectives of this study were to determine the risk factors for PPCs of critical patients admitted to the intensive care unit (ICU) after hepatectomy and create a nomogram model for predicting PPCs.

### Variables and definitions

PPCs were defined according to the Standards for Definitions and use of Outcome Measures for Clinical Effectiveness Research in Perioperative Medicine: European Perioperative Clinical Outcome (EPCO) definitions, which included respiratory infection, respiratory failure, pleural effusion, atelectasis, pneumothorax, bronchospasm, and aspiration pneumonitis [13]; PPCs were defined as the development of one or more of these conditions.

All complications were diagnosed by chest radiography and/or computed tomographic (CT) scan. An experienced intensivist and a radiologist assessed the postoperative respiratory status of all patients. The respiratory infection was defined by chest radiography and/or CT scan results associated with fever and hyperleukocytosis. Respiratory failure was defined as postoperative PaO_2_ < 8 kPa (60 mmHg) in room air, a PaO_2_:FIO_2_ ratio < 40 kPa (300 mmHg), and requiring oxygen therapy. Pleural effusion was defined as chest radiograph findings of blunting of the costophrenic angle or a hazy opacity in one hemithorax with preserved vascular shadows in the supine position. Atelectasis was defined as lung opacification with a shift of the mediastinum, hilum, or hemidiaphragm toward the affected area, and compensatory over-inflation in the adjacent non-atelectatic lung. Pneumothorax was defined as air in the pleural space with no vascular bed surrounding the visceral pleura. Bronchospasm was defined as newly detected expiratory wheezing treated with bronchodilators. Aspiration pneumonitis was defined as acute lung injury after the inhalation of regurgitated gastric contents.

### Data collection

We collected demographic data including sex, age, and body mass index (BMI). Preoperative variables included tobacco use, history of hepatectomy, comorbidities (diabetes, hypertension, coronary heart disease, chronic renal insufficiency), underlying liver disease (hepatitis B virus infection, cirrhosis, portal hypertension, splenomegaly, ascites, steatosis), and laboratory examination (preoperative serum albumin level, postoperative creatine kinase MB [CK-MB] and postoperative brain natriuretic peptide [BNP]). Intraoperative variables included liver lesion (benign/malignant), surgery characteristics (tumor size, tumor number, bilateral subcostal incision, hemihepatectomy, drainage tube, T-tube drainage, operative time, laparotomy, microwave ablation, portal triad clamping period, associated extrahepatic procedures) and anesthetic characteristics (general anesthesia method, intraoperative hypotension, temperature, intraoperative infusion volume, blood loss, urine volume, and nerve block), intraoperative or postoperative blood transfusion and ICU first day infusion volume. Intraoperative hypotension was defined as requiring continuous intraoperative pumping of vasoactive drugs, including norepinephrine, dopamine, and phenylephrine [14, 15]. Two intensivist verified data to avoid bias.

### Statistical analysis

#### Primary statistical analysis

Continuous variables were analyzed using the Student’s t-test or Mann–Whitney U test, as appropriate, and data were expressed as mean values ± SD or median (interquartile range). Categorical variables were analyzed using the chi-squared test or Fisher’s exact test, as appropriate, the n (%) was used to express the data. Statistical tests were done with R software (version 4.0.3; R Foundation for Statistical Computing, Vienna, Austria) and SPSS (version 23.0; SPSS, IBM). A P-value < 0.05 was considered significant.

#### Model development

Univariable logistic regression analyses were performed to assess the association of predictive factors with PPCs. Multivariate analysis was conducted to derive the nomogram. The predictors included in the multivariable model were selected based on the result of univaribable logistic regression analyses (P<0.1)。The backward procedure for variable selection was applied for the multivariable logistic regression model. Regression coefficients were used to generate a nomogram.

#### Predictive ability

Nomogram model performance was assessed by examining discrimination and calibration. The discrimination was assessed by the area under the receiver-operator characteristic and area under curve (AUC) and its 95% confidence interval [CI]. The calibration was constructed to examine the agreement between the predicted probabilities with the observed outcome, which was assessed by the Hosmer–Lemeshow goodness-of-fit test and calibration curve. The calibration curve was calculated by the 500 repetitions bootstrap resampling.

## Results

### Patients

Between January 2014 and January December 2021, 579 patients were admitted to the ICU after hepatectomy, and 503 were finally analyzed (Fig. 1). Data on these patients were recorded in the electronic database on the server and analyzed retrospectively. The rate of PPC occurrence was 55.7% (n = 280). The rates of overall pleural effusion, respiratory failure, atelectasis, respiratory infection, pneumothorax, aspiration pneumonitis, and bronchospasm were 42.5% (n = 214), 31.7% (n = 159), 22.5% (n = 113), 11.1% (n = 56), 0.4% (n = 2), 0.2% (n = 1), and 0% (n = 0), respectively. The distribution of PPCs is shown in Table 1. And for the 159 respiratory failure patients, there are 47 patients with prolonged tracheal intubation duration(more than 24 h), 11 patients with subsequent non-invasive ventilator assistance, and 70 patients with prolonged ICU stay (more than 48 h).

**Table 1:** Distribution of PPCs

Type of PPCs	N (%)
Pleural effusion	214(42.5)
Respiratory failure	159(31.7)
Atelectasis	113(22.5)
Respiratory infection	56(11.1)
Pneumothorax	2(0.4)
Aspiration pneumonitis	1(0.2)
Bronchospasm	0(0)

PPCs: postoperative pulmonary complications

Among the patients, there were 342 men (68.0%) and 161 women (32.0%), the patients had a mean age of 60.3 ± 12.0 years. The underlying liver disease included 193 patients (38.4%) with hepatitis B, 120 patients (23.9%) with cirrhosis, 58 patients (11.5%) with portal hypertension, 71 patients (14.1%) with splenomegaly, 67 patients (13.3%) with ascites, and 14 patients (2.9%) with steatosis. Malignant disease was observed in 87.1% patients (n = 438) and benign disease in 12.9% (n = 65).

The overall mortality rate was 1.6% (n = 8). The causes of death were multiorgan failure (n = 3), gastrointestinal bleeding (n = 1), liver failure (n = 1), heart failure (n = 1), aspiration pneumonia (n = 1), and respiratory distress (n = 1).

### Postoperative pulmonary complications

The mortality rate of patients with PPCs was higher than patients with no PPCs (2.9% vs. 0%, P = 0.011). Patients who developed PPCs had a statistically significant increase in the length of hospital stay (18 [15, 25] days vs. 15 [12, 19] days; P < 0.001), length of ICU stay (2 [2, 4] days vs. 2 [2] days; P < 0.001), and duration of mechanical ventilation (8.0 [4.0, 15.0] hours vs. 5.0 [3.2, 9.5] hours; P < 0.001) (Table 2).

**Table 2:** Outcomes

Outcome	PPCs N = 280	Non-PPCs N = 223	P
In-hospital mortality, n (%)	8 (2.9)	0 (0)	0.011
Length of ICU stay, (IQR days)	2 (2, 4)	2 (2, 2)	< 0.001
Length of hospital stay, (IQR days)	18 (15, 25)	15 (12, 19)	< 0.001
Duration of mechanical ventilation, (IQR hours)	8.0 (4.0, 15)	5.0 (3.0, 9.5)	< 0.001

PPCs: postoperative pulmonary complications. IQR: inter quartile range

The results of univariate analysis are summarized in Table 3. Advanced age, higher BMI, diabetes, hypertension, lower preoperative serum albumin level, postoperative CK-MB, malignant liver lesion, bilateral subcostal incision, prolonged surgery, associated extrahepatic procedures, intraoperative hypotension, greater intraoperative infusion volume, blood loss, urine volume, intra- or postoperative blood transfusion and the ICU first day infusion volume were risk factors for PPCs.

**Table 3:** Univariate analysis of characteristics between the two groups

Characteristic	PPCs N = 280	Non-PPCs N = 223	P
Gender			0.647
Male	188	154	
Female	92	69	
Age, (mean ± SD), years	61.67 ± 11.47	58.47 ± 12.41	0.003
BMI, (mean ± SD), kg/m2	25.13 ± 3.77	23.7 ± 3.41	< 0.001
Tobacco use, n (%)	65 (23.21)	40 (17.93)	0.149
History of hepatectomy, n (%)	45 (16.1)	29 (13.0)	0.327
Comorbidities			
Diabetes, n (%)	68 (24.3)	31 (13.9)	0.004
Hypertension, n (%)	113 (40.5)	55 (24.8)	< 0.001
Coronary heart disease, n (%)	23 (8.2)	20 (9.0)	0.764
Chronic renal insufficiency, n (%)	9 (3.2)	3 (1.3)	0.186
Underlying liver disease			
HBV infection, n (%)	111 (39.6)	82 (36.8)	0.511
Cirrhosis, n (%)	73 (26.1)	47 (21.3)	0.318
Portal hypertension, n (%)	32 (11.4)	26 (11.7)	0.690
Splenomegaly, n (%)	39 (13.9)	32 (14.3)	0.893
Ascites, n (%)	38 (13.6)	29 (13.0)	0.853
Steatosis, n (%)	6 (2.1)	8 (3.6)	0.333
Laboratory examination			
Preoperative serum albumin level, (mean ± SD), g/L	39.09 ± 7.63	40.5 ± 4.64	0.020
Postoperative CK-MB	2.9 (1.9, 4.8)	2.4 (1.56, 3.9)	0.027
Postoperative BNP, (IQR pg/ml)	65.25 (35, 119.5)	60.9 (30, 104.4)	0.328
Liver lesion			0.098
Benign	30	35	
Malign	250	188	
Surgery characteristics			
Tumor size, (IQR cm)	6 (3.3, 10)	6 (4, 10)	0.986
Tumor number ≥ 2, n(%)	118 (42.1)	86 (38.6)	0.417
Bilateral subcostal incision	54 (19.3)	57 (25.6)	0.093
Hemihepatectomy, n(%)	88 (31.4)	74 (33.2)	0.676
Drainage tube, (IQR)	1 (1, 2)	1 (1, 2)	0.701
T-tube drainage, n(%)	34 (12.2)	21 (9.5)	0.326
Operative time, (IQR min)	320 (250, 420)	290 (225, 360)	< 0.001
Laparotomy, n (%)	263 (93.9)	204 (91.5)	0.292
Microwave ablation, n (%)	75 (26.8)	54 (24.2)	0.512
Portal triad clamping period, (IQR min)	0 (0, 12)	0 (0, 12)	0.212
Associated extrahepatic procedures*, n (%)	106 (37.9)	60 (26.9)	0.010
Anesthetic characteristics			
General anesthesia method			0.376
Total intravenous anesthesia	27	27	
Combined intravenous inhalational anesthesia	253	196	
Intraoperative hypotension**, n (%)	62 (22.2)	27 (12.1)	0.004
Temperature, (mean ± SD),°C	35.86 ± 2.23	35.92 ± 0.91	0.723
Intraoperative infusion volume, (IQR L)	4.81 (3.50, 7.28)	4.20 (3.10, 5.70)	< 0.001
Blood loss, (IQR L)	1.28 (0.51, 2.40)	1.00 (0.50, 1.80)	0.009
Urine volume, (IQR L)	0.80 (0.55, 1.30)	0.75 (0.45, 1.05)	0.012
Nerve block, n (%)	31 (11.1)	32 (14.4)	0.263
Intraoperative or postoperative blood transfusion, n (%)	221 (78.9)	150 (67.3)	0.003
ICU first day infusion volume, (IQR L)	6.91 (5.25, 9.47)	5.86 (4.59, 7.75)	< 0.001

Data are expressed as mean ± SD, median (interquartile range), or number (%). PPCs: postoperative pulmonary complications, IQR: inter quartile range, BMI: body mass index, CK-MB: creatine kinase MB, BNP: brain natriuretic peptide. * ≥1 associated procedures in the same patient. **Vasoactive drugs were pumped intraoperatively to maintain blood pressure

**Fig. 2:**
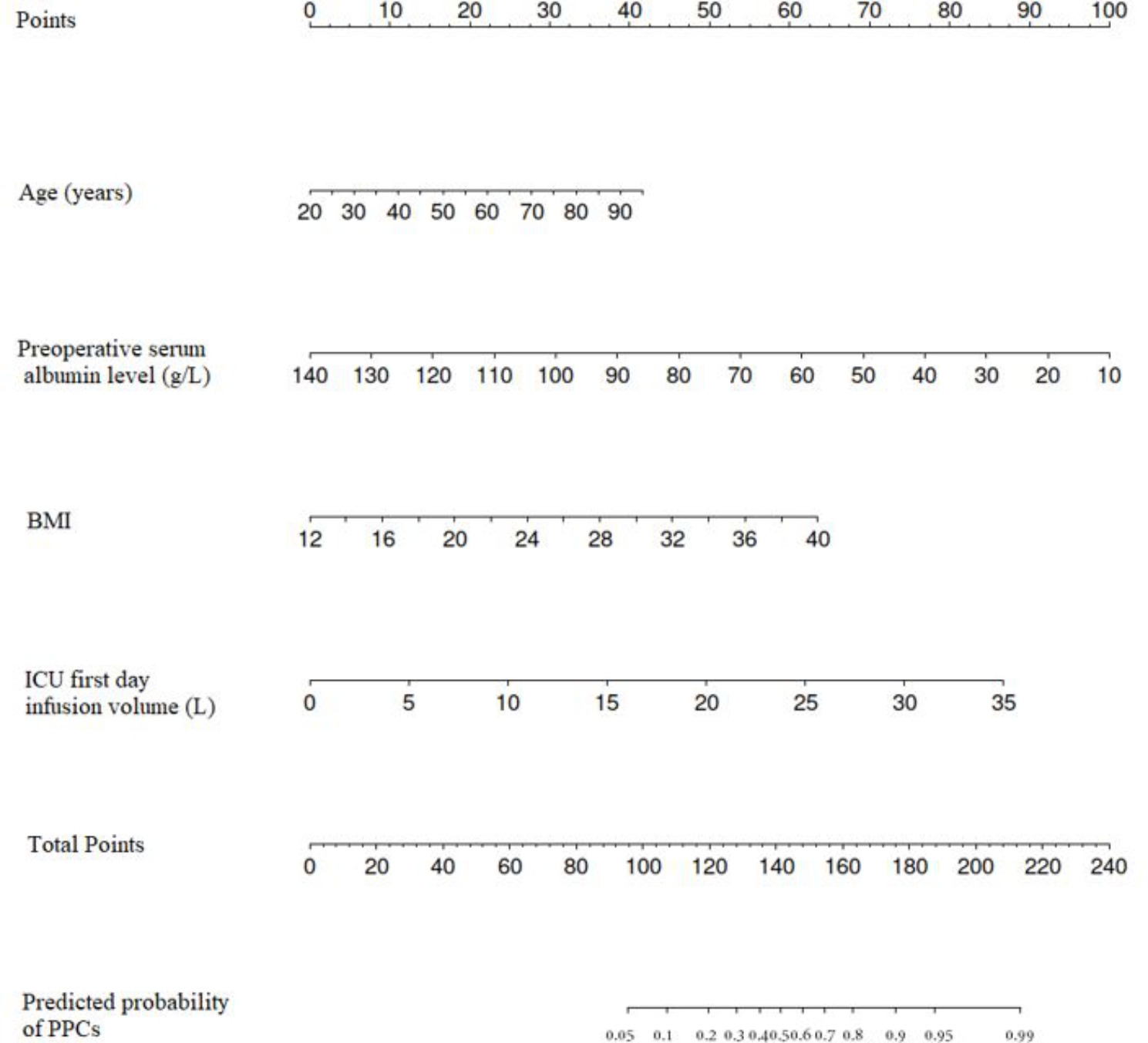
Nomogram for occurrence of PPCs. To estimate the probability of PPCs, mark patient values at each axis, draw a straight line perpendicular to the point axis, and sum the points for all variables. Next, mark the sum on the total point axis and draw a straight line perpendicular to the probability axis. BMI, Body Mass Index. PPCs, Postoperative pulmonary complications

**Fig. 3:**
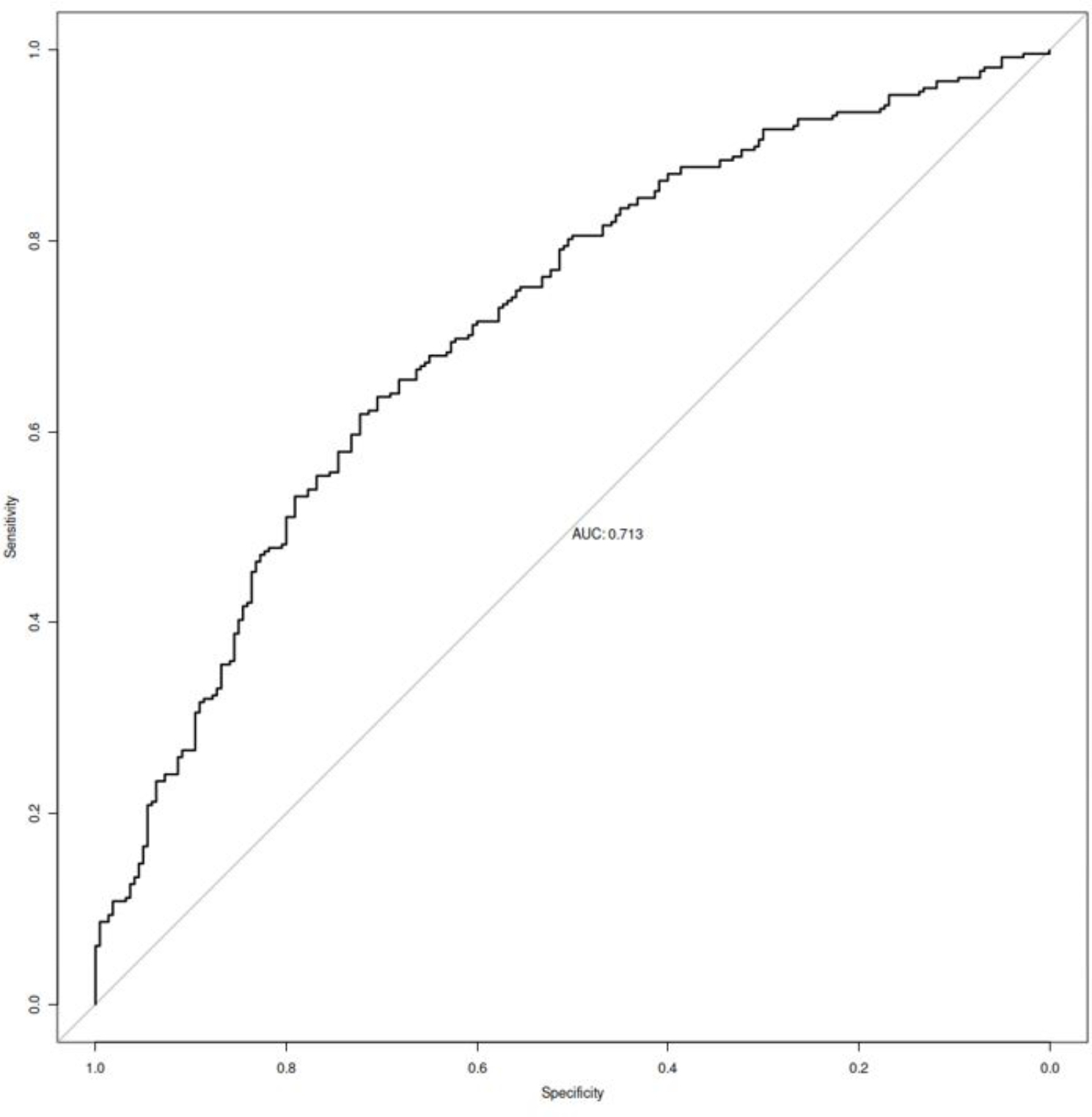
Receiver operating characteristic curve for the prediction model Area under the curve was 0.713 (95% confidence interval 0.668–0.758)

**Fig. 4:**
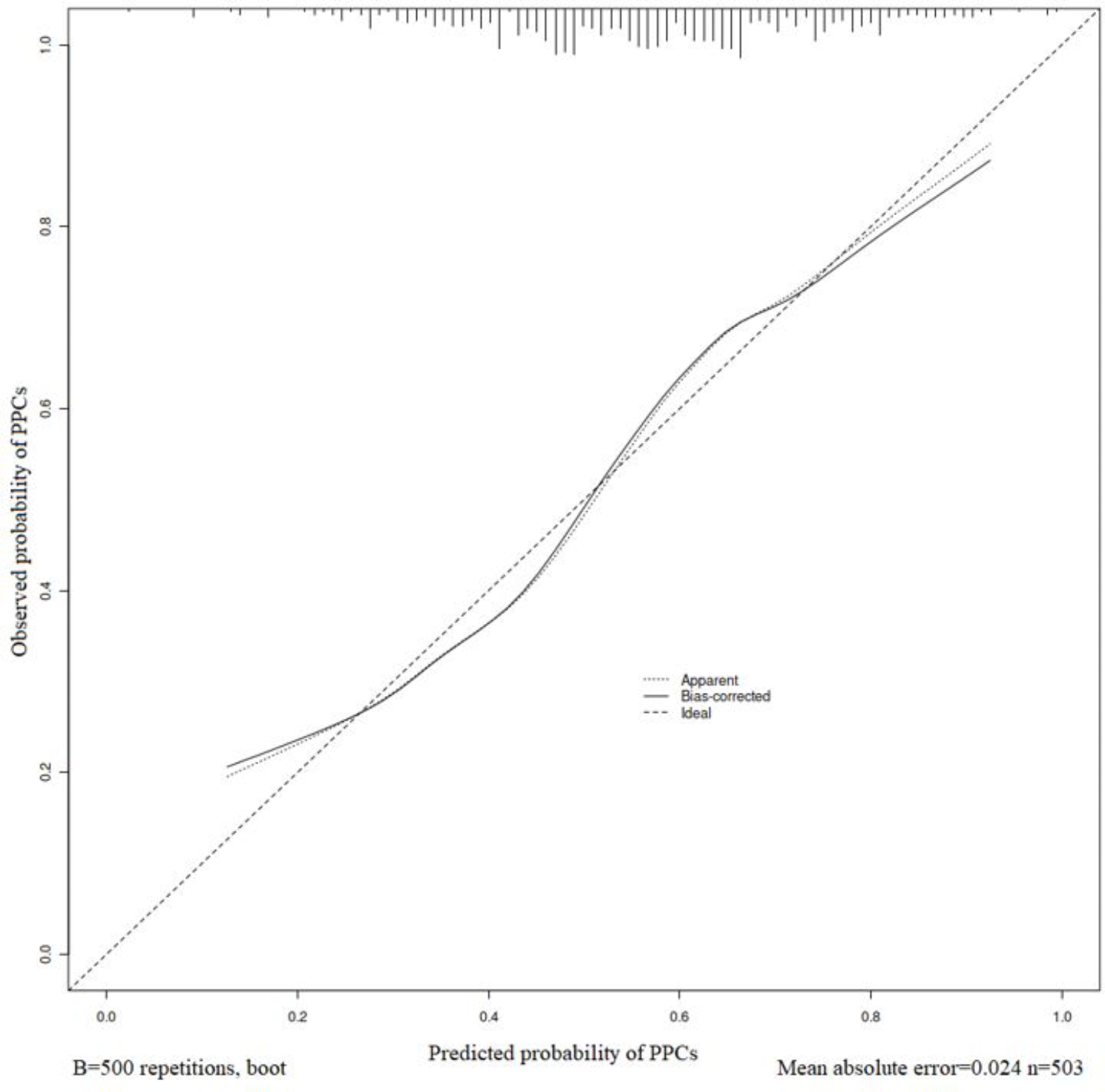
Calibration of the nomogram for PPCs. The x-axis shows the predicted probability of PPCs, and the y-axis shows the observed probability of PPCs. PPCs, Postoperative pulmonary complications

Multivariate analysis identified the following four independent risk factors for PPCs (Table 4): Advanced age (OR = 1.026; P = 0.008), higher BMI (OR = 1.139; P < 0.001), lower preoperative serum albumin level (OR = 0.961; P = 0.037), and ICU first day infusion (OR = 1.152; P = 0.040). And we created a nomogram for PPCs by using these factors (Fig. 2). The area under the curve (AUC) was 0.713 (95% confidence interval 0.668–0.758; Fig. 3). The nomogram had a bootstrapped concordance index of 0.713 and was well calibrated (Fig. 4).

**Table 4:** Risk factors for PPCs: multivariate analysis

Variable	β	Wald	OR	[95% CI]	P
Age	0.025	7.057	1.026	1.007–1.045	0.008
BMI	0.130	17.843	1.139	1.072–1.210	0.000
Diabetes	0.296	1.210	1.344	0.794–2.277	0.271
Hypertension	0.462	3.763	1.588	0.995–2.533	0.052
Preoperative serum albumin level	-0.040	4.368	0.961	0.925-0.998	0.037
Postoperative CK-MB	-0.014	0.127	0.986	0.915–1.064	0.721
Liver lesion	0.259	0.697	1.296	0.705–2.379	0.404
Bilateral subcostal incision	-0.215	0.812	0.806	0.505–1.288	0.367
Operative time	0.002	1.644	1.002	0.999–1.004	0.200
Associated extrahepatic procedures	0.350	2.493	1.419	0.919–2.191	0.114
Intraoperative hypotension	0.418	2.019	1.519	0.853–2.703	0.155
Intraoperative infusion volume	0.004	0.007	1.004	0.907–1.112	0.933
Blood loss	-0.107	1.099	0.899	0.736–1.098	0.295
Urine volume	0.069	0.129	1.072	0.734–1.566	0.720
Intraoperative or postoperative blood transfusion	0.268	1.090	1.308	0.790–2.164	0.296
ICU first day infusion volume	0.142	4.204	1.152	1.006–1.319	0.040

PPCs: postoperative pulmonary complications, OR: Odds ratio, BMI: Body mass index, CK-MB: creatine kinase MB. Odds ratios show the relative incremental risk of each complication when the predictive factor is present. Logistic regression provides 95% confidence intervals as an estimation of relative risk in the general population

## Discussion

Several studies have reported on PPCs after hepatic surgery. However, to the best of our knowledge, no high-quality multivariate analysis has been published on a group of critical patients after hepatectomy in a large population and create a nomogram model for prediction of PPCs. And in our study, we use a clear and rigorous definition to define the PPCs [13].

In this retrospective study, we observed that PPCs occurred in 55.7% of the critical patients admitted in the ICU after hepatectomy. The rates of overall pleural effusion, respiratory failure, atelectasis, respiratory infection, pneumothorax, aspiration pneumonitis, and bronchospasm were 42.5% (n = 214), 31.7% (n = 159), 22.5% (n = 113), 11.1% (n = 56), 0.4% (n = 2), 0.2% (n = 1), and 0% (n = 0), respectively. The incidence of PPCs in our study was higher than that in previous studies [8, 16, 17], which is due to the fact that all patients in our study were critical, and their overall situation after surgery was more complicated. The presence of PPCs was significantly associated with increased postoperative mortality, longer mechanical ventilation time, and prolonged length of stay (LOS) in the ICU and hospital.

In this study, we identified four independent risk factors of PPCs, including advanced age, higher BMI, lower preoperative serum albumin level, and ICU first day infusion volume.

Advanced age as a predictor of PPCs has been proven by previous studies. Several studies [18, 19] have found that age > 60 or 65 years is a risk factor for PPCs, which is in agreement with our finding that age was positively correlated with the occurrence of PPCs. In the elderly, elastic fibers around alveoli and capillaries gradually decrease, and the lung tissue elasticity weakens and the retractive ability decreases. Moreover, arteriosclerosis occurs, vascular lumens become thinner, and the number of capillary networks decreases, resulting in reduced pulmonary blood flow, and reduced effective exchange area of the respiratory membrane, all of which greatly weaken lung function in the elderly. However, some studies have also suggested that frailty may be closely related to PPCs in the elderly, and frailty may be a confounding factor between age and PPCs. Future research could further attempt to differentiate frail and non-frail patients in the elderly.

There is mixed evidence about whether higher BMI is a risk of PPCs in the previous literature. Although some studies have shown that obesity conferred a protective factor against PPCs [20, 21], they stress that BMI does not consider an individual’s body size, and that chest size is more predictive than absolute BMI. In our study, increasing BMI was found to be predictive of PPCs. Obesity causes decreased chest wall compliance, decreased lung volumes, increased oxygen consumption, and increased airway resistance [22–24]. As our study focused on critical patients, the impact of obesity is even more pronounced.

Hypoalbuminemia was also found to be a risk factor for PPCs in our study, which is in agreement with several previous studies [25–27]. The serum albumin level represents the patients’ general nutritional status and their liver reserve function [28], and albumin is responsible for various biological functions. Decreased albumin concentrations inhibit the activation of macrophages, impair the immune response, and increase susceptibility to infection [29]. Besides, hypoalbuminemia reduces plasma colloid osmotic pressure, causing pulmonary edema and pleural effusion and making the lungs susceptible to infection [23, 30].

ICU first day infusion volume in our study means the 24 h infusion volume of the operative day. On one hand, the infusion volume often corelated with the surgery trauma and the hypotension. Larger trauma would cause the severer inflammatory response that would trigger more complications of the patients. And the hypotension is followed by tissue hypoperfusion, which increases postoperative morbidity and mortality [31, 32]. On the other hand, the infusion volume is associated with the clinician’s cognition and habit of the fluid management and resuscitation. More fluid infusion often associated with the more serious edema that would give rise to the pulmonary edema and the abdominal edema and swelling, eventually, these lead to PPCs. And limited fluid resuscitation has gained more international recognition in recent years [33]. This study also supports the clinical implementation of restricted fluid resuscitation.

Risk factors are generally divided into two categories: modifiable factors and unmodifiable factors. In our study, the modifiable factors include preoperative serum albumin level and ICU first day infusion while the unmodifiable factors include age and BMI. To reduce the PPCs, a more aggressive human albumin infusion before surgery to elevate the preoperative serum albumin level may be beneficial and the restricted fluid management and resuscitation would help to reduce the PPCs [34–36]. However, all these findings need to be further verified by conducting large-scale prospective randomized controlled trials.

Meanwhile, we create a nomogram to predict the occurrence of PPCs after hepatectomy in critical patients. The nomogram is composed of age, BMI, preoperative serum albumin level and ICU first day infusion volume, and can be fully utilized soon after surgery. When patients with higher probability of PPCs identified by the nomogram, they should be monitored more carefully during the postoperative period. More aggressive lung revascularization, protein supplementation, and fluid restriction may be helpful. To the best of our knowledge, until now there is no study has build a nomogram for PPCs in these patients after hepatectomy especially in the critical patients.

This study has several limitations. First, this is a single-center retrospective study and the lack of reliable information about pulmonary functional tests did not allow precise assessment of the severity of the underlying pulmonary disease. Second, as the sample size of the study was modest, a lack of power is plausible. Third, for our nomogram external validation is required and furthermore, this model would better be developed into both a user-friendly web-based decision aid platform to assist clinicians.

## Conclusion

PPCs which are common after hepatectomy, were found to be significantly associated with increased postoperative mortality, longer mechanical ventilation time, and prolonged LOS in the ICU and hospital. Advanced age, higher BMI, lower preoperative serum albumin, and the ICU first day infusion volume were independent risk factors for PPCs in critical patients after hepatectomy. And we created a nomogram model which can be used to predict the occurrence of PPCs.

## Data Availability

The datasets used during the current study available from the corresponding author on reasonable request.
